# Epigenome-wide association study (EWAS) on lipids: the Rotterdam Study

**DOI:** 10.1186/s13148-016-0304-4

**Published:** 2017-02-07

**Authors:** Kim V. E. Braun, Klodian Dhana, Paul S. de Vries, Trudy Voortman, Joyce B. J. van Meurs, Andre G. Uitterlinden, BIOS consortium, Albert Hofman, Frank B. Hu, Oscar H. Franco, Abbas Dehghan

**Affiliations:** 1 Department of Epidemiology, Erasmus University Medical Center, Dr. Molewaterplein 50, Office NA2906, PO Box 2040, 3000 CA Rotterdam, The Netherlands; 2 Human Genetics Center, University of Texas Health Science Center at Houston, Houston, TX USA; 3 Department of Internal Medicine, Erasmus University Medical Center, Rotterdam, The Netherlands; 4 The Netherlands Genomics Initiative-sponsored Netherlands Consortium for Healthy Aging (NGI-NCHA), Leiden/Rotterdam, The Netherlands; 5 Department of Epidemiology, Harvard T.H. Chan School of Public Health, Boston, MA USA; 6 Department of Nutrition, Harvard T.H. Chan School of Public Health, Boston, MA USA

**Keywords:** DNA methylation, Lipids, EWAS, Triglycerides, HDL-C, LDL-C, Total cholesterol, Cohort

## Abstract

**Background:**

DNA methylation is a key epigenetic mechanism that is suggested to be associated with blood lipid levels. We aimed to identify CpG sites at which DNA methylation levels are associated with blood levels of triglycerides, high-density lipoprotein cholesterol (HDL-C), low-density lipoprotein cholesterol (LDL-C), and total cholesterol in 725 participants of the Rotterdam Study, a population-based cohort study. Subsequently, we sought replication in a non-overlapping set of 760 participants.

**Results:**

Genome-wide methylation levels were measured in whole blood using the Illumina Methylation 450 array. Associations between lipid levels and DNA methylation beta values were examined using linear mixed-effect models. All models were adjusted for sex, age, smoking, white blood cell proportions, array number, and position on array. A Bonferroni-corrected *p* value lower than 1.08 × 10^−7^ was considered statistically significant. Five CpG sites annotated to genes including *DHCR24*, *CPT1A*, *ABCG1*, and *SREBF1* were identified and replicated. Four CpG sites were associated with triglycerides, including CpG sites annotated to *CPT1A* (cg00574958 and cg17058475), *ABCG1* (cg06500161), and *SREBF1* (cg11024682). Two CpG sites were associated with HDL-C, including *ABCG1* (cg06500161) and *DHCR24* (cg17901584). No significant associations were observed with LDL-C or total cholesterol.

**Conclusions:**

We report an association of HDL-C levels with methylation of a CpG site near *DHCR24*, a protein-coding gene involved in cholesterol biosynthesis, which has previously been reported to be associated with other metabolic traits. Furthermore, we confirmed previously reported associations of methylation of CpG sites within *CPT1A*, *ABCG1*, and *SREBF1* and lipids. These results provide insight in the mechanisms that are involved in lipid metabolism.

**Electronic supplementary material:**

The online version of this article (doi:10.1186/s13148-016-0304-4) contains supplementary material, which is available to authorized users.

## Background

Genetics is an important determinant of lipid levels which may affect them by changing the expression levels of the genes [1]. Gene expression levels, however, are also regulated by DNA methylation, which is one of the most studied mechanisms in the field of epigenetics and may therefore have an effect on lipid levels [2]. In contrast to the DNA sequence, DNA methylation is dynamic over time and responsive to the environment; therefore, DNA methylation could also change in response to blood lipid levels [3].

A few studies using candidate gene approaches have reported that DNA methylation at several loci, such as *APOE* and *ABCA1*, are associated with lipid levels [4, 5]. In addition, epigenome-wide association studies (EWAS) have recently become available, providing the possibility to identify associations between blood lipid levels and DNA methylation at novel loci [3]. To date, EWAS have identified associations between differentially methylated genes at a few novel loci, such as *TNNT1*, *CPT1A*, and *ABCG1*, and blood lipid levels [6–8]. However, so far, most studies investigating the association between DNA methylation and lipids have been performed in patient populations, while only one study has been performed within a population-based study. As DNA methylation may vary across different states of health, further population-based studies are needed to explore these associations in the general population.

In this study, we aimed to investigate the association between blood DNA methylation levels and blood levels of triglycerides, high-density lipoprotein cholesterol (HDL-C), low-density lipoprotein cholesterol (LDL-C), and total cholesterol in of 725 participants of the Rotterdam Study, a population-based cohort study. Subsequently, we sought replication in a non-overlapping set of 760 participants.

## Results

### Participant characteristics

Participant characteristics of the discovery cohort (*n* = 725) and the replication cohort (*n* = 760) are presented in Table 1. Levels of triglycerides and total cholesterol were similar in both cohorts. Mean levels of HDL were slightly lower in the discovery cohort compared to the replication cohort (1.4 vs. 1.5 mmol/L, *p* < 0.001). Mean levels of LDL-C were higher in the discovery cohort compared to the replication cohort (3.9 vs. 3.7 mmol/L, *p* = 0.02). The mean age was significantly higher (*p* < 0.001) in the replication cohort (67.7 ± 5.9 years) compared to the discovery cohort (59.9 ± 8.2 years). In the discovery cohort, 27% of the population was current smokers, whereas in the replication cohort, this was 10% (*p* < 0.001).

**Table 1:** Participant characteristics

	Discovery^a^	Replication^a^	*p* value^b^
*N*	725	760	
Gender (male)	336 (46%)	324 (42%)	0.14
Age (years)	59.9 ± 8.2	67.7 (5.9)	<0.001
BMI (kg/m^2^)	27.6 ± 4.6	27.8 (4.2)	0.48
Obesity (BMI > 30)	173 (24%)	194 (26%)	0.54
Waist circumference	93.7 ± 12.8	94.4 ± 12.0	0.318
Current smoking (yes)	197 (27%)	79 (10%)	<0.001
Triglycerides (mmol/L)	1.3 [0.9–1.8]	1.3 [1.0–1.7]	0.66
HDL-cholesterol (mmol/L)	1.4 (0.41)	1.5 (0.44)	<0.001
LDL-cholesterol (mmol/L)	3.9 (1.00)	3.7 (0.95)	0.001
Total cholesterol (mmol/L)	5.6 (1.07)	5.5 (1.03)	0.26
Lipid-lowering medication (yes)	191 (26%)	238 (31%)	<0.001
CHD	42 (6%)	61 (8%)	0.003
DM	72 (10%)	94 (12%)	0.13

^a^Values are presented as mean ± SD, median [IQR], or *N* (%)

^b^Characteristics of the discovery cohort and replication cohort were compared with ANOVA

### Discovery panel

**Fig. 1:**
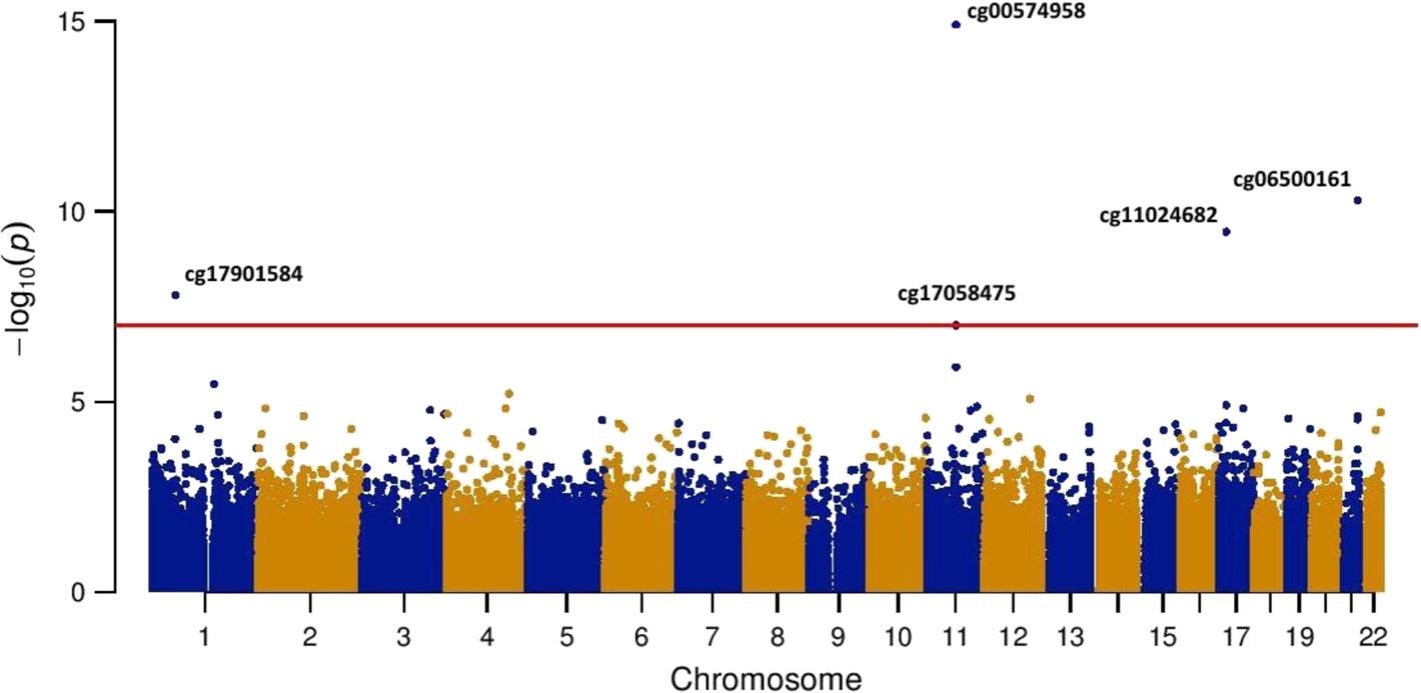
Manhattan plot epigenome-wide associations between genome-wide DNA methylation and triglycerides

**Fig. 2:**
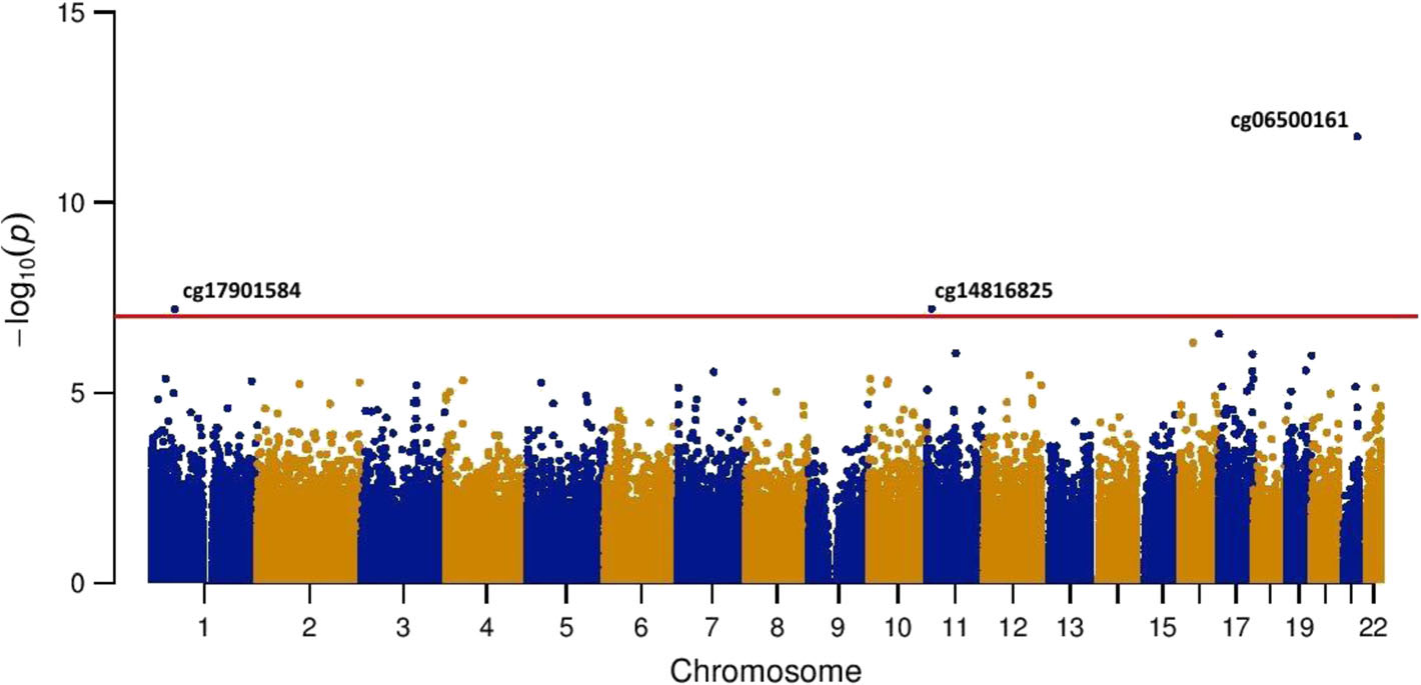
Manhattan plot epigenome-wide associations between genome-wide DNA methylation and HDL

The associations between DNA methylation probes and blood lipid levels are presented in Manhattan plots (Figs. 1 and 2). Table 2 shows the Bonferroni-significant CpGs. We identified five CpG sites associated with triglyceride levels. These CpG sites were annotated to *CPT1A* (cg00574958 and cg17058475), *ABCG1* (cg06500161), *SREBF1* (cg11024682), and *DHCR24* (cg17901584). CpG sites annotated to *CPT1A* and *DHCR24* were negatively associated with triglycerides, whereas CpG sites annotated to *ABCG1* and *SREBF1* were positively associated with triglycerides. We identified three CpG sites associated with HDL-C. These CpG sites were annotated to *ABCG1* (cg06500161) and *DHCR24* (cg17901584), and one CpG site was not annotated to a gene (cg14816825). The CpG site annotated to *DHCR24* was positively associated with HDL-C; the other two CpG sites were negatively associated with HDL-C. We did not find significantly associated CpG sites for LDL-C and total cholesterol levels in the discovery cohort.

**Table 2:** Epigenome-wide associations between genome-wide DNA methylation and lipid levels

				Discovery		Replication		
	CpG site	Chromosome	Position	Regression coefficient	*p* ^a^	Regression coefficient	*p* ^a^	Gene
Triglycerides	cg00574958	11	68607622	−0.0206	*1.23 × 10* ^*−15*^	−0.0114	*7.24 × 10* ^*−13*^	*CPT1A*
	cg06500161	21	43656587	0.0149	*5.02 × 10* ^*−11*^	0.0201	*1.46 × 10* ^*−15*^	*ABCG1*
	cg11024682	17	17730094	0.0135	*3.40 × 10* ^*−10*^	0.0172	*3.27 × 10* ^*−11*^	*SREBF1*
	cg17901584	1	55353706	−0.0162	*1.56 × 10* ^*−08*^	−0.0068	9.54 × 10^−02^	*DHCR24*
	cg17058475	11	68607737	−0.0183	*9.84 × 10* ^*−08*^	−0.0118	*1.71 × 10* ^*−06*^	*CPT1A*
HDL-C	cg06500161	21	43656587	−0.0202	*1.84 × 10* ^*−12*^	−0.0189	*3.29 × 10* ^*−12*^	*ABCG1*
	cg14816825	11	12128203	−0.0143	*6.31 × 10* ^*−08*^	−0.0051	1.15 × 10^−01^	*NA* ^b^
	cg17901584	1	55353706	0.0196	*6.44 × 10* ^*−08*^	0.0142	*1.03 × 10* ^*−03*^	*DHCR24*

Models are adjusted for age, gender, current smoking, leukocyte proportions, array number, and position on array

Values are regression coefficients based on linear mixed models and reflect differences in methylation beta values per increase in HDL-C or log-transformed triglyceride unit

^a^ *P* values in italic indicate statistical significance. Level of significance: *p* < 1.08 × 10^−07^(discovery cohort), *p* < 7.14 × 10^−03^(replication)

^b^Not annotated

### Replication panel

Of the five CpG sites significantly associated with triglycerides in the discovery cohort, four replicated in the replication cohort, including *CPT1A* (cg00574958 and cg17058475), *ABCG1* (cg06500161), and *SREBF1* (cg11024682). Of the three CpG sites significantly associated with HDL-C in the discovery cohort, two replicated in the replication cohort, including *ABCG1* (cg06500161) and *DHCR24* (cg17901584). Results from the discovery and replication cohorts were combined using fixed-effect meta-analyses.

### Meta-analyses

In order to test potential confounding, additional models with adjustment for lipid-lowering medication, waist circumference, and other lipids were performed in the combined analyses. Some of the effect estimates decreased in strength, but overall, the results remained similar to those of model 1 (Table 3). Furthermore, results from meta-analyses revealed seven new CpG sites associated with triglycerides, including *TXNIP*, *TMEM49*, *SLC7A11*, and *KCNA3*. An additional 55 CpG sites were associated with HDL-C. Methylation of four CpG sites were associated with total cholesterol, including CpGs annotated to *IFFO1*, *ABCG1*, and *DHCR24* (Additional file 1: Table S1).

**Table 3:** Associations between genome-wide DNA methylation and lipid levels (meta-analyses)

		Model 2^a^		Model 3^b^		Model 4^c^		
	ProbeID	Regression coefficient	*p* ^d^	Regression coefficient	*p* ^d^	Regression coefficient	*p* ^d^	Gene
TG	cg00574958	−0.0142	*2.6 × 10* ^*−26*^	−0.0114	*1.9 × 10* ^*−16*^	−0.0161	*1.5 × 10* ^*−22*^	*CPT1A*
	cg06500161	0.0167	*1.4 × 10* ^*−24*^	0.0153	*3.9 × 10* ^*−18*^	0.0151	*7.0 × 10* ^*−14*^	*ABCG1*
	cg11024682	0.0147	*3.0 × 10* ^*−19*^	0.0124	*8.4 × 10* ^*−13*^	0.0138	*1.0 × 10* ^*−11*^	*SREBF1*
	cg17058475	−0.0146	*3.1 × 10* ^*−13*^	−0.0120	*8.8 × 10* ^*−09*^	−0.0196	*9.2 × 10* ^*−16*^	*CPT1A*
	cg17901584	−0.013	*1.9 × 10* ^*−08*^	−0.0104	*2.5 × 10* ^*−05*^	−0.012	*2.5 × 10* ^*−05*^	*DHCR24*
HDL-C	cg06500161	−0.0187	*9.5 × 10* ^*−23*^	−0.0166	*9.5 × 10* ^*−16*^	−0.0116	*2.5 × 10* ^*−07*^	*ABCG1*
	cg14816825	−0.0104	*3.2 × 10* ^*−07*^	−0.0145	*3.8 × 10* ^*−07*^	−0.0127	*1.7 × 10* ^*−07*^	*NA* ^e^
	cg17901584	0.0164	*2.4 × 10* ^*−09*^	0.0128	*1.3 × 10* ^*−05*^	0.0116	*3.6 × 10* ^*−04*^	*DHCR24*

All models are adjusted for age, gender, current smoking, leukocyte proportions, array number, and position on array

Values are regression coefficients based on linear mixed models and reflect differences in methylation beta values per increase in HDL-C or log-transformed triglyceride unit

^a^Model 2: model 1 + lipid-lowering medication use

^b^Model 3: model 1 + waist circumference

^c^Model 4: model 1 + other lipids

^d^ *P* values in italic indicate statistical significance. Level of significance: *p* < 7.14 × 10^−03^

^e^Not annotated

### Additional analyses

Since lipid levels might be affected by dietary fat intake, we explored whether intake of total fat, poly-unsaturated fatty acids (PUFA), mono-unsaturated fatty acids (MUFA), and saturated fatty acids (SFA) were associated with DNA methylation of significantly replicated CpG sites, using linear regression models. From these models, we observed no significant association between fat intake and methylation of *DHCR24*, *SREBF1*, *ABCG1*, or *CPT1A* (Additional file 2: Table S2). To test if there was an interaction between lipid-lowering medication or fat intake and the CpG site located in the *DHCR24* gene on blood lipid levels, interaction terms were added to the regression model. However, none of these interaction terms were significant. Sensitivity analyses in which we replaced beta values with M values showed similar results (Additional file 3: Table S3).

### Methylation risk scores

**Fig. 3:**
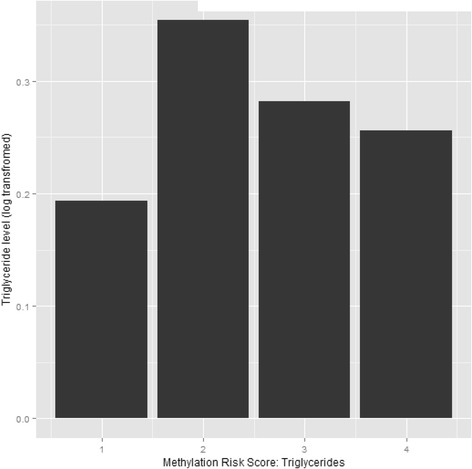
Methylation risk score in quartiles and levels of triglycerides

**Fig. 4:**
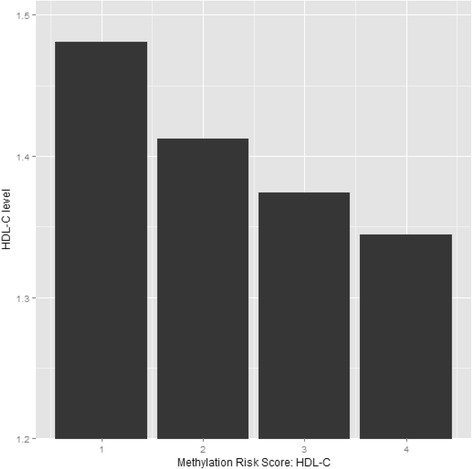
Methylation risk score in quartiles and levels of HDL-C

The methylation risk score was calculated using eight CpG sites for triglycerides and seven CpG sites for HDL-C, based on current and previously reported findings [7, 8]. The correlation coefficients of these CpG sites are presented in Additional file 4: Table S4 and Additional file 5: Table S5. For triglycerides, 9% of the variance was explained by the methylation risk score. For HDL-C, 5% of the variance was explained by the methylation risk score (Additional file 6: Table S6). To test whether the association between methylation risk scores and lipids differed by lipid-lowering medication use, interaction terms were tested. For both triglycerides as HDL-C, none of the interaction terms were significant. The difference in levels of triglycerides and HDL-C per quartile of methylation risk score are presented in Figs. 3 and 4. HDL-C levels decrease as quartiles of methylation risk score increase. Triglyceride levels increased from the first quartile to the second quartile but remained similar for the third quartile and the fourth quartile.

### Expression levels

The associations between DNA methylation of *ABCG1* and *CPT1A* and their expression levels are presented in Additional file 7: Table S7. Of the four genes of which the significantly associated CpG sites were annotated, only data on expression levels for *CPT1A* and *ABCG1* passed quality control. For *CPT1A*, the expression (ILMN_1696316) was negatively associated with the methylation of *CPT1A* at both identified CpG sites (cg00574958: *p* = 7.6 × 10^−4^, cg17058475: *p* = 5.1 × 10^−3^). Expression of *ABCG1* at two of the five transcripts (ILMN_1794782 and ILMN_2329927) were negatively associated with methylation of the *ABCG1* CpG site (*p* = 6.6 × 10^−14^ and 3.7 × 10^−11^).

## Discussion

The current study reports the results of an epigenome-wide association study of fasting triglycerides, HDL-C, LDL-C, and total cholesterol blood levels. We identified and replicated a CpG site near *DHCR24* to be related with HDL-cholesterol. Moreover, we confirmed former reports that methylation of CpG sites within *ABCG1* are associated with HDL-C, and methylation of CpG sites within *CPT1A*, *ABCG1*, and *SREBF1* were associated with triglycerides. The majority of these associations were independent of several potentially confounding factors, such as use of lipid-lowering medication, waist circumference, or other lipid traits. The differentially methylated CpG sites combined in a methylation risk score explained up to 9% of the variance in triglycerides and 5% of the variance in HDL-C.

**Fig. 5:**
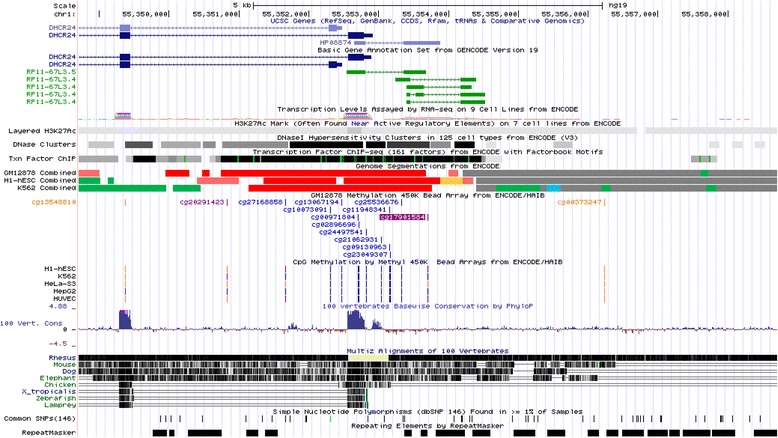
Annotation of cg17901584 to *DHCR24*

We observed that methylation of a CpG site (cg17901584) annotated to the *DHCR24* gene was associated with HDL-C. This CpG site is located within 1 Kb upstream of the *DHCR24* gene (Fig. 5). The *DHCR24* gene encodes for the cholesterol biosynthesis enzyme 3-hydroxysterol-24 reductase, which catalyzes the conversion of desmosterol to cholesterol [9, 10]. Mutations in the *DHCR24* gene may cause desmosterolosis, an autosomal recessive disease characterized by high levels of desmosterol [11]. Since *DHCR24* is involved in the cholesterol metabolism, an association between methylation in this gene and HDL-C is plausible. However, to date, it is not clear how methylation of *DHCR24* is involved in sterol regulation. It has been suggested that regulation of *DHCR24* expression is mediated by sterol regulatory element-binding proteins (SREBP) in response to cholesterol availability [12]. We observed in meta-analysis of the discovery and replication cohort that methylation of a CpG site in the *SREBF1* gene, which encodes for *SREBP*, was associated with HDL-C levels. These findings suggest that mechanisms in which these two genes are involved might interact. Unfortunately, as the probes for these genes did not pass quality control, we did not have data on the expression levels of these genes to further investigate this hypothesis. Moreover, the *DHCR24* gene is located near *PCSK9*, which is associated with cholesterol levels [1]. Therefore, the association we observed between *DHCR24* methylation and HDL-C might be due to SNP variation in *PCSK9*. However, when we adjusted our models on HDL-C for the top SNP from genome-wide association studies (GWAS), the association remained similar (data not shown). To our knowledge, an association between methylation of cg17901584 (*DHCR24*) and HDL-C has not been previously reported. However, an EWAS performed by Dekkers et al. showed that another CpG site located in the *DHCR24* gene, cg2716885, was associated with LDL-C levels [13]. Although there are no previous studies that report an association between methylation of cg17901584 (*DHCR24*) and HDL-C, previous studies reported associations between methylation of this CpG site with waist circumference and phosphatidylcholine (PC ae C36:5) [14, 15]. In additional models, we adjusted for waist circumference, where we indeed observed a decrease in strength compared to the main model. However, the association stayed significant. Two scenarios may explain these results. A higher waist circumference may affect lipid levels and consequently modify DNA methylation. Alternatively, waist circumference could be a confounding factor since metabolic traits are highly correlated. Due to the cross-sectional design of our study, it is difficult to make strong conclusions whether waist circumference is a confounder or a precursor in this association.

In addition to our novel finding, we also replicated findings previously reported by the GOLDN study [7]. In line with findings from GOLDN, we observed a significant negative association between two CpG sites (cg00574958 and cg17058475) located in the *CPT1A* gene and triglycerides. Carnitine palmitoyltransferase-1, which is encoded by the *CPT1A* gene, has an important role of transporting long-chain fatty acids in mitochondria. In order to obtain more knowledge on the epigenetic mechanisms of *CPT1A*, we explored expression levels of this gene in relation to methylation. Similar to findings from the GOLDN study, we observed a negative association between DNA methylation and expression levels of one probe, suggesting that increased methylation at *CPT1A* decreases expression of this gene [7].

Furthermore, some of the findings previously reported by KORA were also replicated [8]. In line with findings from KORA, we also found a significant negative association between a CpG site (cg06500161), located in the *ABCG1* gene and HDL-C. We also found a significant positive association between CpG sites located in *ABCG1* (cg06500161) and *SREBF1* (cg11024682) and triglycerides. Although these findings regarding triglycerides correspond to findings from KORA, the CpG sites in *ABCG1* and *SREBF1* differed from the CpG sites found in our study. To further explore the epigenetic mechanisms of *ABCG1*, we investigated whether DNA methylation of the CpG sites at *ABCG1* was associated with gene expression. Similar to KORA, we observed a negative correlation between DNA methylation at CpG sites annotated to *ABCG1* with two mRNA expression probes [8]. In our study, ILMN_1794782 showed the strongest negative correlation (*r* = −0.30) with methylation of *ABCG1* (cg06500161), which was also reported by the authors from KORA as one of the most strongly related transcripts to *ABCG1* methylation. These results are in agreement with a mediatory role of gene *ABCG1* expression in the association between DNA methylation and lipid levels. In addition to lipid levels, other studies have also demonstrated an association between methylation levels at CpG sites located in *CPT1A*, *ABCG1*, and *SREBF1* with other cardiometabolic traits, such as myocardial infarction, body mass index (BMI), waist circumference, insulin, and several metabolomic traits [8, 14–16]. These previously reported findings and our current results suggest that methylation of these genes are involved in metabolic mechanisms and may be potential therapeutic targets for cardiovascular- and metabolic-related diseases.

Results from the discovery and replication cohorts were combined in meta-analysis, which revealed a large amount of additional differentially methylated CpG sites that are associated with HDL-C levels. These additional findings include several CpG sites annotated to genes that were previously reported in GWAS on lipid levels, such as *LDLR* and *CMIP* [1]. *LDLR*, low-density lipoprotein receptor, is a protein-coding gene, and mutations in this gene can cause familial hypercholesterolemia [17]. *CMIP* encodes a c-Maf-inducing protein, which is involved in T cell signaling pathway, and this gene is known to be associated with speech impairment [18]. However, less is known on the role of *CMIP* in cholesterol metabolism. Considering previously reported and current findings, there might be a genetic effect as well as an epigenetic effect of these genes on HDL-C levels. Nevertheless, these findings should be interpreted with caution, as the results of our meta-analyses are not replicated by other studies yet.

This study has several strengths and limitations that should be considered with the interpretation of the currently reported results. The strength of this study is the availability of genome-wide DNA methylation data in a large sample of adults from the general population and an internal replication set. Although we had a large sample size, more loci can be identified with even larger sample sizes. To illustrate, when the results from the discovery and replication cohorts were combined in meta-analysis, an additional set of 55 new significant associations for HDL-C were observed (Additional file 1: Table S1), which emphasizes the benefits of using a larger sample size in EWAS. A limitation of this study is the use of whole blood samples to determine DNA methylation levels, whereas DNA methylation is cell type specific. When methylation is studied as a consequence of lipid levels, then leukocytes are a relevant tissue. However, when the aim is to study methylation sites that are causal of lipid levels, leukocytes might not be a relevant tissue. Therefore, certain important CpG sites could have been overlooked in our study. Nevertheless, previous studies have demonstrated that results can be replicated across tissues, suggesting that the use of blood tissue is not necessarily a major issue [14, 19]. Moreover, associations may have been overlooked be due to the type of measurements used in our study. The levels of LDL-C in our study were calculated with use of the Friedewald formula [20], which may not have been specific enough to identify an association between genome-wide DNA methylation and LDL-C. Results from another study performed within the GOLDN study showed that methylation of two CpG sites in the *CPT1A* gene is associated with LDL-C and VLDL-C [21]. These results overlap with our findings of triglycerides, but we did not identify CpG sites associated with LDL. This discrepancy in results may be due to a more detailed quantification of lipids by NMR spectroscopy in the GOLDN study. In our current study, we used beta values to analyze methylation levels. Since it has been suggested that there could be heteroscedasticity for CpG sites with very low or high methylation, the use of M values is recommended. These M values represent the Logit transformation of the beta values and are considered to have a better detection rate and true positive rate [22]. In order to explore whether this could have affected our results, we performed additional analyses in which we replaced beta values with M values. However, these sensitivity analyses showed that results were similar for beta values and M values (Additional file 3: Table S3), which suggests that in our study heteroscedasticity may not be an issue. Due to the cross-sectional design, we cannot determine the temporal direction of the association between DNA methylation and blood lipids. Furthermore, the observed associations could be explained by an unidentified common factor, as residual confounding is always an issue in observational studies. Another possibility is that the results may be confounded by differences in cell type proportion. In order to avoid this source of confounding, we adjusted all analyses for measured or estimated cell type proportions [23]. Finally, our replication cohort had a 7.8 years higher mean age compared to the discovery cohort, which could have resulted in differences in association between DNA methylation and lipid levels. However, when we tested our models for interaction with age, we did not find any evidence that the strength of associations was affected by age.

## Conclusion

In conclusion, we report an association of HDL-C levels with methylation of a CpG site near *DHCR24*, a protein-coding gene involved in cholesterol biosynthesis. This CpG site has previously been reported to be associated with other metabolic traits, such as waist circumference. In addition, we replicated associations previously reported by other studies. These results provide insight in the mechanisms that are involved in cholesterol and lipid metabolism and identify potential new therapeutic targets. Future studies should include a larger sample size and further investigate the independency and causality of the observed associations.

## Methods

### Design and subjects

This study was embedded within the Rotterdam Study, a population-based cohort study in Rotterdam, the Netherlands. The design of the Rotterdam Study has been previously described in detail elsewhere [24]. Briefly, residents of Ommoord, a district in Rotterdam, aged 45 years and older were invited to participate. The Rotterdam Study includes three sub-cohorts. We used data from the baseline and second visit of the third cohort (RSIII-1 and RSIII-2) and the third visit from the second cohort (RSII-3).

### Discovery panel

We used the data from RSIII-1 as the discovery panel: between February 2006 and December 2008, 3932 participants were examined. EWAS measurements were performed on a random subset of 731 subjects, of whom 725 had fasting blood samples available and were included in the current analyses.

### Replication panel

We sought replication in a set of 767 participants from RSII-3 and RSIII-2. Between February 2011 and February 2012, 1887 participants from RSII-3 were examined. Between March 2012 and December 2013, approximately 3000 participants from RSIII-2 were examined. From the participants included in the replication study, 760 had fasting blood samples available and were included for analyses. None of the participants included in the replication study were included in the discovery cohort.

### DNA methylation

DNA was extracted from whole blood (stored in EDTA tubes) by standardized salting out methods. Genome-wide methylation levels were measured using the Illumina Infinium HumanMethylation450 Beadchip (Illumina Inc., San Diego, CA) [25]. Briefly, samples (500 ng of DNA per sample) were bisulfite treated with use of the Zymo EZ-96 DNA methylation kit (Zymo Research, Irvine, CA, USA). Thereafter, the samples were hybridized to the arrays according to the protocol of the manufacturer. During quality control in RSIII-1, samples showing incomplete bisulfite treatment were excluded (*n* = 5) as were samples with a low detection rate (<99%) (*n* = 7) and gender swaps (*n* = 4). Probes with a detection *p* value >0.01 in >1% of the samples were filtered out [26, 27]. In RSII-3 and RSIII-2, outlying samples were checked using the first two principal components obtained using principal component analysis (PCA). None of the samples failed the quality control checks, indicating high quality data. Per individual probe, participants with methylation levels higher than three times the inter-quartiles range (IQR) were excluded. The methylation proportion of a CpG site was reported as a beta value ranging from 0 to 1 [28]. We used the genome coordinates provided by Illumina (GRCh37/hg19) to identify independent loci.

### mRNA expression data

Total RNA was isolated (PAXgene Blood RNA kits—Qiagen) from whole blood (PAXgene Tubes—Becton Dickinson). All RNA samples were analyzed using the LabChip GX (Caliper) according to the manufacturer’s instructions, to ensure a constant high quality of RNA preparations. Samples with an RNA quality score >7 were amplified and labeled (Ambion TotalPrep RNA) and hybridized to the Illumina HumanHT-12 v4 Expression BeadChips, as described by the manufacturer’s protocol. RNA samples were processed at the Genetic Laboratory of Internal Medicine, Erasmus University Medical Center Rotterdam. The dataset including 881 expression samples from RSIII-1 is available at GEO (Gene Expression Omnibus) public repository under the accession GSE338828. Gene expression data was quantile normalized to the median distribution and log2 transformed. Probe and sample means were centered to zero. Genes were considered significantly expressed when detection *p* values calculated by Genome Studio were less than 0.05 in more than 10% of all discovery samples, which added to a total number of 21,238 probes. The eQTL-mapping pipeline was used to perform quality control [29].

### Blood lipids

All participants had blood samples taken during the visits to the research center. From the blood samples, concentrations of triglycerides, HDL-C, and total cholesterol were measured using an automated enzymatic method. LDL-C was calculated using the Friedewald formula (total cholesterol − HDL-C − triglycerides/5) [20]. Participants with non-fasting blood samples were excluded from the current analyses (*n* = 6).

### Covariates

Height and weight were measured during the center visit, and BMI was calculated (kg/m^2^). During home visit interviews, data on tobacco smoking, dietary intake, and medication use were collected. Information on smoking history was acquired from questionnaires and categorized as never, former, or current smoking. Nutritional data was collected using semi-quantitative FFQs, and information on the intake of different types of fatty acids were obtained. Fat intake was reported as total fat, PUFA, MUFA, and SFA. Information regarding the use of lipid-lowering medication was derived from both structured home interviews and linkage to pharmacy records.

### Statistical analysis

Triglyceride level was log transformed using a natural log to obtain a normal distribution. The associations between lipid levels and DNA methylation beta values were examined using linear mixed-effect models.

### Discovery

All models were adjusted for sex, age, smoking (current, former, or never), white blood cell proportions, and technical covariates (model 1). Gender, age, and smoking were added to the model as fixed effects. To correct for cell mixture distribution, leukocyte proportions (CD8+ T cells, CD4+ T cells, NK cells, B cells, monocytes, and granulocytes) were estimated using the Houseman method and were added to the model as fixed effects [23]. Technical covariates included array number and position on the array, and these were added to the models as random effects. To account for multiple testing, we used a Bonferroni-corrected *p* value of 1.08 × 10^−7^ (0.05/463,456 probes).

### Replication

Identified probes were replicated using the same models as in the discovery cohort, further adjusted for the cohort. The adjustment for cell counts (monocytes, granulocytes, and lymphocytes) was based on lab measurements rather than Houseman estimates. For the replication, we applied a Bonferroni-corrected significance threshold of 6.25 × 10^−3^ (0.05/8 probes).

### Meta-analyses

To combine results from the discovery and replication cohorts, fixed-effect meta-analyses were performed in METAL, using an inverse variance weighted method. In subsequent analyses, models were further adjusted for lipid-lowering medication use (model 2), waist circumference (model 3), and other lipids (model 4).

### Additional analyses

Lipid levels may be affected by dietary fat intake, and this might be mediated through DNA methylation. Therefore, we explored associations between different types of fatty acid intake and DNA methylation at significantly replicated CpG sites. To account for potential measurement error and confounding by total energy intake, we used the residual method to adjust the fatty acid intake for total energy intake. Briefly, linear regression analyses were used with energy intake as the independent variable and fatty acid intake as the dependent variable to calculate the energy-adjusted intake of individual fatty acids for each subject. We regressed out the estimated leukocyte proportions, age, sex, array number, and position on array on the beta values of the CpG sites using linear mixed models. The associations between energy-adjusted fatty acid intakes and the residuals of the DNA methylation beta values were examined using linear regression models. All models were adjusted for sex, age, total energy intake (kcal/day), and smoking. Furthermore, to test if there was an interaction between lipid-lowering medication or fat intake and methylation at novel loci on lipids, interaction terms were added to the regression model, with one of the lipid traits as the outcome. In post hoc analyses, we adjusted our models on HDL-C for the *PCSK9* genotype, as *DHCR24* and *PCSK9* are located near each other. In these analyses, the top SNP from GWAS (rs17111503) was added to our main model (model 1). As it is recommended to use M values due to heteroscedasticity in beta values, we performed additional analyses in which we replaced beta values with M values for comparison [22].

### Methylation risk score

A methylation risk score was calculated based on CpG sites that were associated with the phenotypes, using both newly identified CpG sites in the current study as well as the ones previously reported for the corresponding trait [7, 8]. First, CpG sites were checked for correlation and CpGs were pruned giving priority to the most significant CpGs reported by the largest studies using a correlation coefficient cutoff of 0.6. Second, linear regression analyses were performed in the replication cohort, using the lipids as the dependent variable and the included CpG sites as independent variables. Models were adjusted for age, sex, blood cell counts, and technical covariates. The effect estimates were used to build the methylation risk score using data from the discovery panel. With the use of linear regression models, we calculated the lipid variance explained by the methylation risk score.

### Functional analyses

Considering that DNA methylation can have an effect on the expression of genes, we explored the association between DNA methylation at the statistically significant CpG sites identified by EWAS and mRNA expression of the corresponding genes. The DNA methylation proportions and mRNA expression levels of these genes were checked for association using linear mixed models, which were adjusted for age, sex, smoking, white blood cell proportions, and technical covariates (array number and position on array).

## Additional files

Additional file 1: Table S1. Statistically significant associations in meta-analyses from the discovery and replication cohorts between genome-wide DNA methylation and lipid levels. (DOCX 22 kb)
Additional file 2: Table S2. Associations between DNA methylation at significant CpG sites and fatty acid intake. (DOCX 14 kb)
Additional file 3: Table S3. Statistically significant associations from the discovery cohort between lipid levels and genome-wide DNA methylation. Beta values compared to M values. (DOCX 13 kb)
Additional file 4: Table S4. Correlations between CpG sites included in the triglyceride methylation risk score. (DOCX 14 kb)
Additional file 5: Table S5. Correlations between CpG sites included in the HDL-C methylation risk score. (DOCX 14 kb)
Additional file 6: Table S6. Lipid variation explained by methylation risk score, age, and sex. (DOCX 14 kb)
Additional file 7: Table S7. Correlations between DNA methylation at significant CpG sites and expression probes. (DOCX 14 kb)

## Abbreviations

BMI: Body mass index
EWAS: Epigenome-wide association study
GWAS: Genome-wide association studies
HDL-C: High-density lipoprotein cholesterol
LDL-C: Low-density lipoprotein cholesterol
RS: Rotterdam Study
