# Supplementary material for: Epigenome-wide association study (EWAS) on lipids: the Rotterdam Study
Source: Clin Epigenetics. 2017 Feb 7;9:15. doi: 10.1186/s13148-016-0304-4 (PMC5297218; doi:10.1186/s13148-016-0304-4)
Supplement: Additional file 1: Table S1. — Statistically significant associations in meta-analyses from the discovery and replication cohorts between genome-wide DNA methylation and lipid levels. (DOCX 22 kb) [file 13148_2016_304_MOESM1_ESM.docx]

**Table S1. Statistically significant associations in meta-analyses from discovery and replication cohort between genome wide DNA methylation and lipid levels.**

|  | **ProbeID** | **chr** | **pos** | **Effect** | **P** | **Gene** |
| --- | --- | --- | --- | --- | --- | --- |
| **Triglycerides** | cg00574958 | 11 | 68607622 | -0.0139 | 5.07×10^-26^ | *CPT1A* |
|  | cg06500161 | 21 | 43656587 | 0.0173 | 1.42×10^-25^ | *ABCG1* |
|  | cg11024682 | 17 | 17730094 | 0.0150 | 2.85×10^-20^ | *SREBF1* |
|  | cg17901584 | 1 | 55353706 | -0.0131 | 1.52×10^-08^ | *DHCR24* |
|  | cg17058475 | 11 | 68607737 | -0.0140 | 1.53×10^-12^ | *CPT1A* |
|  | cg19693031 | 1 | 145441552 | -0.0133 | 6.72×10^-08^ | *TXNIP* |
|  | cg24174557 | 17 | 57903544 | -0.0145 | 7.00×10^-10^ | *TMEM49* |
|  | cg06690548 | 4 | 139162808 | -0.0089 | 3.91×10^-08^ | *SLC7A11* |
|  | cg27243685 | 21 | 43642366 | 0.0085 | 9.90×10^-15^ | *ABCG1* |
|  | cg01176028 | 21 | 43653234 | 0.0123 | 1.22×10^-08^ | *ABCG1* |
|  | cg13925011 | 1 | 111216387 | -0.0097 | 1.26×10^-08^ | *KCNA3* |
|  | cg06734985 | 11 | 26849501 | -0.0129 | 5.19×10^-08^ | *NA^3^* |
|  |  |  |  |  |  |  |
| **HDL-C** | cg06500161 | 21 | 43656587 | -0.0195 | 4.16×10^-24^ | *ABCG1* |
|  | cg17901584 | 1 | 55353706 | 0.0170 | 5.78×10^-10^ | *DHCR24* |
|  | cg06017212 | 17 | 1478463 | -0.0109 | 1.67×10^-09^ | *SLC43A2* |
|  | cg11969813 | 17 | 79816559 | -0.0161 | 2.04×10^-08^ | *P4HB* |
|  | cg21088259 | 17 | 81039990 | -0.0121 | 4.92×10^-09^ | *METRNL* |
|  | cg01101459 | 1 | 234871477 | -0.0137 | 1.21×10^-09^ | *NA^3^* |
|  | cg14468090 | 3 | 129032864 | -0.0110 | 2.96×10^-10^ | *NA^3^* |
|  | cg26313301 | 19 | 11219615 | -0.0056 | 3.28×10^-08^ | *LDLR* |
|  | cg08174890 | 4 | 9558077 | 0.0088 | 3.74×10^-09^ | *MIR548I2* |
|  | cg03031932 | 16 | 81547138 | -0.0106 | 2.82×10^-11^ | *CMIP* |
|  | cg02578470 | 3 | 127320670 | -0.0141 | 2.53×10^-09^ | *MCM2* |
|  | cg07730360 | 3 | 128845626 | -0.0110 | 2.91×10^-09^ | *NA^3^* |
|  | cg08132940 | 7 | 1081526 | -0.0160 | 2.46×10^-08^ | *C7orf50* |
|  | cg27243685 | 21 | 43642366 | -0.0105 | 2.79×10^-16^ | *ABCG1* |
|  | cg11024682 | 17 | 17730094 | -0.0137 | 1.10×10^-12^ | *SREBF1* |
|  | cg07091481 | 10 | 82169149 | -0.0131 | 5.26×10^-08^ | *C10orf58* |
|  | cg07691624 | 3 | 9886198 | -0.0083 | 7.04×10^-09^ | *RPUSD3* |
|  | cg14939082 | 10 | 104535990 | -0.0135 | 2.22×10^-08^ | *C10orf26* |
|  | cg00521255 | 5 | 139726689 | -0.0116 | 2.82×10^-08^ | *HBEGF* |
|  | cg20214535 | 21 | 43619310 | -0.0099 | 8.45×10^-08^ | *ABCG1* |
|  | cg07565956 | 17 | 7381288 | -0.0102 | 1.21×10^-08^ | *ZBTB4* |
|  | cg20605134 | 6 | 15400462 | -0.0104 | 8.01×10^-08^ | *JARID2* |
|  | cg25217710 | 1 | 156609523 | -0.0086 | 1.87×10^-08^ | *NA^3^* |
|  | cg00805360 | 10 | 135091210 | -0.0142 | 2.49×10^-10^ | *ADAM8* |
|  | cg13876650 | 1 | 26146005 | -0.0085 | 1.30E×10^-08^ | *FAM54B* |
|  | cg02797539 | 17 | 72740524 | -0.0109 | 4.02×10^-08^ | *RAB37* |
|  | cg06372475 | 10 | 73534286 | -0.0080 | 6.08×10^-08^ | *C10orf54* |
|  | cg25877299 | 17 | 80273290 | -0.0142 | 4.26×10^-09^ | *CD7* |
|  | cg04262505 | 2 | 217850623 | 0.0088 | 8.55×10^-08^ | *NA^3^* |
|  | cg08352115 | 17 | 66356057 | -0.0097 | 7.93×10^-08^ | *ARSG* |
|  | cg21113318 | 14 | 92983645 | -0.0096 | 9.72×10^-09^ | *RIN3* |
|  | cg26943120 | 4 | 5472116 | -0.0213 | 3.60×10^-09^ | *STK32B* |
|  | cg11849692 | 10 | 103875969 | -0.0099 | 3.48×10^-08^ | *LDB1* |
|  | cg03074946 | 12 | 120687694 | -0.0122 | 2.74×10^-09^ | *PXN* |
|  | cg27269962 | 7 | 127540997 | -0.0082 | 4.50×10^-08^ | *SND1* |
|  | cg09473207 | 6 | 155079453 | -0.0118 | 8.72×10^-10^ | *RBM16* |
|  | cg07175797 | 16 | 50317656 | -0.0109 | 9.64×10^-11^ | *NA^3^* |
|  | cg27366162 | 17 | 66375195 | -0.0128 | 1.72×10^-08^ | *ARSG* |
|  | cg12179380 | 8 | 142084624 | -0.0104 | 2.45×10^-08^ | *NA^3^* |
|  | cg26468878 | 5 | 112501418 | 0.0098 | 7.36×10^-08^ | *MCC* |
|  | cg09256683 | 17 | 34313637 | 0.0077 | 9.50×10^-08^ | *CCL14* |
|  | cg06151145 | 4 | 48346434 | -0.0136 | 2.44×10^-08^ | *SLAIN2* |
|  | cg06710464 | 17 | 79047695 | -0.0096 | 5.39×10^-08^ | *BAIAP2* |
|  | cg23799393 | 10 | 60588674 | 0.0069 | 6.29×10^-09^ | *BICC1* |
|  | cg04061506 | 2 | 48137261 | -0.0115 | 4.20×10^-08^ | *NA^3^* |
|  | cg05457221 | 10 | 134272437 | -0.0090 | 5.42×10^-08^ | *NA^3^* |
|  | cg26405097 | 6 | 15428301 | -0.0157 | 1.88×10^-09^ | *JARID2* |
|  | cg06876354 | 2 | 121020189 | -0.0100 | 3.57×10^-10^ | *RALB* |
|  | cg00819078 | 5 | 16615081 | -0.0152 | 2.35×10^-18^ | *FAM134B* |
|  | cg10127660 | 8 | 21996234 | -0.0107 | 2.57×10^-08^ | *REEP4* |
|  | cg05045598 | 1 | 6418521 | -0.0123 | 4.58×10^-08^ | *ACOT7* |
|  | cg15483436 | 14 | 105857208 | -0.0068 | 1.23×10^-09^ | *PACS2* |
|  | cg18805734 | 12 | 31453901 | -0.0072 | 4.23×10^-08^ | *FAM60A* |
|  | cg26640901 | 10 | 104536035 | -0.0104 | 4.82×10^-08^ | *C10orf26* |
|  | cg13139542 | 2 | 8242815 | -0.0071 | 1.73×10^-08^ | *NA^3^* |
|  | cg08788930 | 8 | 142201685 | -0.0116 | 8.11×10^-08^ | *DENND3* |
|  |  |  |  |  |  |  |
| **Total cholesterol** | cg17281677 | 12 | 6658557 | -0.0035 | 5.57×10^-08^ | *IFFO1* |
|  | cg25536676 | 1 | 55353327 | 0.0044 | 7.77×10^-08^ | *DHCR24* |
|  | cg06500161 | 21 | 43656587 | -0.0043 | 3.66×10^-08^ | *ABCG1* |
|  | cg17475467 | 1 | 55316769 | -0.0023 | 8.97×10^-08^ | *DHCR24* |

*^1^Models adjusted for age, gender, current smoking, houseman estimated leukocyte proportions, array number, and position on array (model 1).*

*^2^Only hits that are statistically significant (p<1.08×10^-07^) in the combined analyses are shown in the table*

*^3^ Not annotated.*
