# Supplementary material for: Epigenome-wide association study (EWAS) on lipids: the Rotterdam Study
Source: Clin Epigenetics. 2017 Feb 7;9:15. doi: 10.1186/s13148-016-0304-4 (PMC5297218; doi:10.1186/s13148-016-0304-4)
Supplement: Additional file 2: Table S2. — Associations between DNA methylation at significant CpG sites and fatty acid intake. (DOCX 14 kb) [file 13148_2016_304_MOESM2_ESM.docx]

**Table S2. Associations between DNA methylation at significant CpG sites and fatty acid intake.^1^**

|  |  |  |  | | | | |  | |  | |
| --- | --- | --- | --- | --- | --- | --- | --- | --- | --- | --- | --- |
|  | **ProbeID** | **Gene** | | **Regression coefficient** | **P** | |  | | | | |
|  |  |  | |  |  | |  | | | | |
| **Total Fat** | cg17901584 | *DHCR24* | | -0.00002 | 0.78 | |  | | | | |
|  | cg11024682 | *SREBF1* | | -0.00001 | 0.89 | |  | | | | |
|  | cg06500161 | *ABCG1* | | 0.00003 | 0.61 | |  | | | | |
|  | cg00574958 | *CPT1A* | | -0.00011 | 0.10 | |  | | | | |
|  | cg17058475 | *CPT1A* | | -0.00005 | 0.60 | |  | | | | |
|  |  |  | |  |  | |  | | | | |
| **SFA^2^** | cg17901584 | *DHCR24* | | -0.00011 | 0.53 | |  | | | | |
|  | cg11024682 | *SREBF1* | | 0.00003 | 0.80 | |  | | | | |
|  | cg06500161 | *ABCG1* | | 0.00008 | 0.55 | |  | | | | |
|  | cg00574958 | *CPT1A* | | -0.00019 | 0.22 | |  | | | | |
|  | cg17058475 | *CPT1A* | | 0.00024 | 0.28 | |  | | | | |
|  |  |  | |  |  | |  | | | | |
| **MUFA^2^** | cg17901584 | *DHCR24* | | -0.00011 | 0.53 | |  | | | | |
|  | cg11024682 | *SREBF1* | | -0.00009 | 0.48 | |  | | | | |
|  | cg06500161 | *ABCG1* | | 0.00004 | 0.77 | |  | | | | |
|  | cg00574958 | *CPT1A* | | -0.00023 | 0.13 | |  | | | | |
|  | cg17058475 | *CPT1A* | | -0.00019 | 0.38 | |  | | | | |
|  |  |  | |  |  | |  | | | | |
| **PUFA^2^** | cg17901584 | *DHCR24* | | -0.00011 | 0.55 | |  | | | | |
|  | cg11024682 | *SREBF1* | | 0.00000 | 0.98 |  | | |  | |  |
|  | cg06500161 | *ABCG1* | | 0.00003 | 0.82 |  | | |  | |  |
|  | cg00574958 | *CPT1A* | | -0.00018 | 0.29 |  | | |  | |  |
|  | cg17058475 | *CPT1A* | | -0.00036 | 0.13 |  | | |  | |  |
|  |  |  | |  |  |  | | |  | |  |

*Values are regression coefficients based on linear mixed models and reflect differences in methylation beta values per increase in triglycerides or HDL-C unit.*

*^1^Models adjusted for age, gender, current smoking, and total energy intake.*

*^2^PUFA, poly-unsaturated fatty acids; MUFA, mono-unsaturated fatty acids; SFA, saturated fatty acids.*
