# Supplementary material for: Epigenome-wide association study (EWAS) on lipids: the Rotterdam Study
Source: Clin Epigenetics. 2017 Feb 7;9:15. doi: 10.1186/s13148-016-0304-4 (PMC5297218; doi:10.1186/s13148-016-0304-4)
Supplement: Additional file 3: Table S3. — Statistically significant associations from the discovery cohort between lipid levels and genome-wide DNA methylation. Beta values compared to M values. (DOCX 13 kb) [file 13148_2016_304_MOESM3_ESM.docx]

**Table S3. Statistically significant associations from the discovery cohort between lipid levels and genome wide DNA methylation Beta-values compared to M-values.**

|  |  |  |  |  | **M-value^1,2^** |  |
| --- | --- | --- | --- | --- | --- | --- |
|  | **ProbeID** | **chr** | **pos** |  | **Effect^3^** | **P^4^** |
| TG | cg00574958 | 11 | 68607622 |  | -0.1925 | 2.09E×10^-16^ |
|  | cg06500161 | 21 | 43656587 |  | 0.1042 | 1.51E×10^-10^ |
|  | cg11024682 | 17 | 17730094 |  | 0.0799 | 2.96E×10^-10^ |
|  | cg17058475 | 11 | 68607737 |  | -0.1630 | 2.59E×10^-08^ |
|  | cg17901584 | 1 | 55353706 |  | -0.1061 | 2.64E×10^-08^ |
|  |  |  |  |  |  |  |
| HDL-C | cg06500161 | 21 | 43656587 |  | -0.1406 | 7.78E×10^-12^ |
|  | cg14816825 | 11 | 12128203 |  | -0.1123 | 1.48E×10^-07^ |
|  | cg17901584 | 1 | 55353706 |  | 0.1368 | 1.46E×10^-08^ |

*^1^Values are regression coefficients based on linear mixed models and reflect differences in methylation M-values per increase in HDL-C or log transformed triglycerides* *unit.*

*^2^M-value=log2(Beta/(1-Beta))*

*^3^Models are adjusted for age, gender, current smoking, leukocyte proportions, array number, and position on array.*

*^4^Level of significance: p<1.08×10^-07^*
