# Supplementary material for: Epigenome-wide association study (EWAS) on lipids: the Rotterdam Study
Source: Clin Epigenetics. 2017 Feb 7;9:15. doi: 10.1186/s13148-016-0304-4 (PMC5297218; doi:10.1186/s13148-016-0304-4)
Supplement: Additional file 4: Table S4. — Correlations between CpG sites included in the triglyceride methylation risk score. (DOCX 14 kb) [file 13148_2016_304_MOESM4_ESM.docx]

**Table S4. Correlations between CpG sites included in the triglycerides methylation risk score.**

|  | | **cg00574958** | **cg07504977** | **cg20544516** | **cg07397296** | **cg07815238** | | **cg06500161** | | **cg11024682** | |
| --- | --- | --- | --- | --- | --- | --- | --- | --- | --- | --- | --- |
| **cg00574958** | 1 | | -0.07 | -0.03 | -0.04 | | 0.12 | | -0.24 | | -0.15 |
| **cg07504977** | - | | 1 | -0.35 | 0.17 | | -0.35 | | 0.02 | | 0.03 |
| **cg20544516** | - | | - | 1 | 0.14 | | 0.43 | | 0.24 | | 0.30 |
| **cg07397296** | - | | - | - | 1 | | 0.18 | | 0.22 | | 0.15 |
| **cg07815238** | - | | - | - | - | | 1 | | 0.19 | | 0.17 |
| **cg06500161** | - | | - | - | - | | - | | 1 | | 0.32 |
| **cg11024682** | - | | - | - | - | | - | | - | | 1 |

*Correlation coefficients were based on Pearson correlation r^2^*
