# Supplementary material for: Epigenome-wide association study (EWAS) on lipids: the Rotterdam Study
Source: Clin Epigenetics. 2017 Feb 7;9:15. doi: 10.1186/s13148-016-0304-4 (PMC5297218; doi:10.1186/s13148-016-0304-4)
Supplement: Additional file 5: Table S5. — Correlations between CpG sites included in the HDL-C methylation risk score. (DOCX 14 kb) [file 13148_2016_304_MOESM5_ESM.docx]

**Supplemental Table S5. Correlations between CpG sites included in the HDL-C methylation risk score.**

|  | **cg06500161** | **cg19693031** | **cg11024682** | **cg00574958** | **cg27243685** | **cg14816825** | **cg17901584** |
| --- | --- | --- | --- | --- | --- | --- | --- |
| **cg06500161** | 1 | -0.07 | 0.32 | -0.24 | 0.48 | 0.05 | -0.01 |
| **cg19693031** | - | 1 | 0.15 | 0.24 | -0.01 | 0.26 | 0.01 |
| **cg11024682** | - | - | 1 | -0.15 | 0.43 | 0.35 | 0.26 |
| **cg00574958** | - | - | - | 1 | -0.09 | -0.13 | 0.15 |
| **cg27243685** | - | - | - | - | 1 | -0.01 | 0.27 |
| **cg14816825** | - | - | - | - | - | 1 | -0.32 |
| **cg17901584** | - | - | - | - | - | - | 1 |

*Correlation coefficients were based on Pearson correlation r^2^*
