# Supplementary material for: Epigenome-wide association study (EWAS) on lipids: the Rotterdam Study
Source: Clin Epigenetics. 2017 Feb 7;9:15. doi: 10.1186/s13148-016-0304-4 (PMC5297218; doi:10.1186/s13148-016-0304-4)
Supplement: Additional file 6: Table S6. — Lipid variation explained by methylation risk score, age, and sex. (DOCX 14 kb) [file 13148_2016_304_MOESM6_ESM.docx]

**Table S6. Lipid variation explained by methylation risk score, age, and sex.^1^**

|  | Triglycerides | HDL-C |
| --- | --- | --- |
| MRS^2^ | 0.09 | 0.05 |
| Age | 0.002 | 0.001 |
| Sex | 0.02 | 0.17 |
| Age + sex | 0.026 | 0.173 |
| MRS + age + sex | 0.10 | 0.21 |

*^1^Variance explained by the methylation scores (multiple R^2^, adjusting for age and sex) was calculated using a linear regression models*

*^2^MRS, methylation risk score.*
