# Supplementary material for: Epigenome-wide association study (EWAS) on lipids: the Rotterdam Study
Source: Clin Epigenetics. 2017 Feb 7;9:15. doi: 10.1186/s13148-016-0304-4 (PMC5297218; doi:10.1186/s13148-016-0304-4)
Supplement: Additional file 7: Table S7. — Correlations between DNA methylation at significant CpG sites and expression probes. (DOCX 14 kb) [file 13148_2016_304_MOESM7_ESM.docx]

**Table S7. Correlations between DNA methylation at significant CpG sites and expression probes.^1^**

|  |  |  | |  | |  | |  | |  |  |
| --- | --- | --- | --- | --- | --- | --- | --- | --- | --- | --- | --- |
| **CpG site^1^** | **Gene** | | **Location^2^** | | **Expression probe** | | **N** | **Correlation coefficients** | **P** | | |
|  |  | |  | |  | |  |  |  | | |
| cg00574958 | *CPT1A* | | 5’UTR | | ILMN_1696316 | | 713 | -0.12 | 2.0 × 10^-03^ | | |
| cg17058475 | *CPT1A* | | 5’UTR | | ILMN_1696316 | | 712 | -0.09 | 1.4 × 10^-02^ | | |
| cg06500161 | *ABCG1* | | Body | | ILMN_1695968 | | 712 | -0.02 | 5.6 × 10^-01^ | | |
| cg06500161 | *ABCG1* | | Body | | ILMN_1794782 | | 712 | -0.30 | 2.2 × 10^-16^ | | |
| cg06500161 | *ABCG1* | | Body | | ILMN_2262362 | | 712 | -0.06 | 1.1 × 10^-01^ | | |
| cg06500161 | *ABCG1* | | Body | | ILMN_2329927 | | 712 | -0.18 | 1.9 × 10^-06^ | | |
|  |  | |  | |  | |  |  |  | | |

*^1^Correlation coefficients are based on pearson correlation test of methylation beta values and expression probe unit.*

*^2^ Annotation according to genome coordinates provided by Illumina (GRCh37/hg19)*
